# Size-dependent and tunable crystallization of GeSbTe phase-change nanoparticles

**DOI:** 10.1038/srep39546

**Published:** 2016-12-20

**Authors:** Bin Chen, Gert H. ten Brink, George Palasantzas, Bart J. Kooi

**Affiliations:** 1 Zernike Institute for Advanced Materials University of Groningen, Nijenborgh 4, 9747AG Groningen, the Netherlands

## Abstract

Chalcogenide-based nanostructured phase-change materials (PCMs) are considered promising building blocks for non-volatile memory due to their high write and read speeds, high data-storage density, and low power consumption. Top-down fabrication of PCM nanoparticles (NPs), however, often results in damage and deterioration of their useful properties. Gas-phase condensation based on magnetron sputtering offers an attractive and straightforward solution to continuously down-scale the PCMs into sub-lithographic sizes. Here we unprecedentedly present the size dependence of crystallization for Ge_2_Sb_2_Te_5_ (GST) NPs, whose production is currently highly challenging for chemical synthesis or top-down fabrication. Both amorphous and crystalline NPs have been produced with excellent size and composition control with average diameters varying between 8 and 17 nm. The size-dependent crystallization of these NPs was carefully analyzed through *in-situ* heating in a transmission electron microscope, where the crystallization temperatures (*T*_*c*_) decrease when the NPs become smaller. Moreover, methane incorporation has been observed as an effective method to enhance the amorphous phase stability of the NPs. This work therefore elucidates that GST NPs synthesized by gas-phase condensation with tailored properties are promising alternatives in designing phase-change memories constrained by optical lithography limitations.

Phase-change materials (PCMs) have attracted intensive interests, because they offer large optical and electrical contrast between amorphous and crystalline phases in combination with rapid and reversible switching between these two phases and with excellent prospects for down-scaling. This unique set of properties makes PCMs excellently suited for data storage applications (rewriteable optical media and phase-change random-access memory)^1^,^2^,^3^ and future applications, including neuromorphic computing^4^,^5^, flexible displays^6^, logic devices^7^, plasmonic-based circuits^8^, optically reconfigurable metasurfaces and all-photonic devices^9^. Because of the potential advantages, such as ultra-high switching speed and density of data storage^10^,^11^, down-scaling of the PCMs into nanostructured form has evoked intensive explorations, where pronounced size-dependence on physical properties has been revealed, such as size-dependent crystallization and polar ordering^12^,^13^,^14^,^15^. For example, solution based GeTe nanoparticles (NPs) with an average diameter of 1.8 nm display a crystallization temperature (*T*_*c*_) of 400 °C, 220 °C higher than the one for bulk GeTe (~180 °C)^12^,^13^. Furthermore, size-dependent nucleation and activation energy for crystal growth have been observed for Ge_2_Sb_2_Te_5_ (GST) nanowires when their widths are down-scaled to tens of nanometers^15^.

GST, the prototypical PCM, exhibits the fastest phase transition measured in memory devices so far^7^. The technological relevance of scaling memory devices and the scientific interest to understand size-effects on crystallization have stimulated many investigations on three dimensional down-scaling of GST PCMs. GST nanogaps, connected by carbon nanotube electrodes, have been prepared via top-down process, displaying a much lower switching current (two orders of magnitude) than the ‘state-of-the-art’ devices^11^,^16^. Nevertheless it is highly challenging to control the sizes of the phase-change nanogaps (ranging from 20 nm to 300 nm in 100 memories) as they are for instance produced by electrical breakdown of the carbon nanotube. In another work^17^, GST nanodots have been prepared by self-assembled block-copolymers as a patterning template for PCM deposition, where GST nanodots with an average diameter of around 15 nm show an anomalously direct transition from the amorphous to the rhombohedral phase at around 400 °C with some unknown peaks in X-ray diffraction patterns, bypassing the rock-salt phase which is usually formed for more bulk-like GST during heating.

Compared to top-down processing, bottom-up techniques provide remarkably better control in size and shape of the materials^18^. Although solution based synthesis can produce extraordinary GeTe NPs, it remains highly challenging to synthesize other PCMs NPs, such as binary GeSb or even more complicated ternary (pseudo-binary) GeSbTe systems^18^. Laser ablation is another alternative that has been explored to produce GST NPs, yet inconsistent but exceptional results have been reported on the crystallization of GST NPs. The GST NPs prepared by this method were observed to form a rhombohedral phase dominated mixture at lower temperature and to have pure rock-salt structure at higher temperatures. Although the same phase-change sequences are observed, the critical temperatures showed a big discrepancy in these two works (300 and 400 °C^19^ versus 100 and 200 °C^20^, respectively). More importantly, the wide size distribution of these NPs (4–30 nm and 5–25 nm) makes them unsuitable to explore the size-dependent crystallization. From the contradictory results on GST NPs described above, the crystallization of GST NPs remains elusive, in particular concerning the size-dependence of crystallization. More elaborate and systematic investigations are desired to understand size dependence of the amorphous to crystalline transition of GST NPs. Magnetron sputtering based on gas-phase condensation is a one-step and promising solution-free method to produce NPs. The NPs produced by this method are ‘clean’ (without surrounding ligands), enabling to exempt the possible influence from the ligands. The narrow size distributions of the produced NPs enable statistical studies on the size-dependence of crystallization. In this manuscript we present a systematic study of the crystallization of as-deposited amorphous GST NPs with scalable sizes varying with a factor of two. Adopting magnetron sputtering based on inert gas condensation, the lithographic limitations in size are overcome. Size-dependent crystallization has been unprecedentedly observed for these NPs via *in-situ* heating in a transmission electron microscope (TEM). Simultaneously, methane, as an incorporation gas, has been found to remarkably increase the crystallization temperatures, indicating a facile method to enhance the stability of the amorphous GST NPs. This study therefore can facilitate further developments of devices based GST PCMs at sub-lithographic scales.

## Results and Discussion

### Control of crystallinity

**Figure 1:**
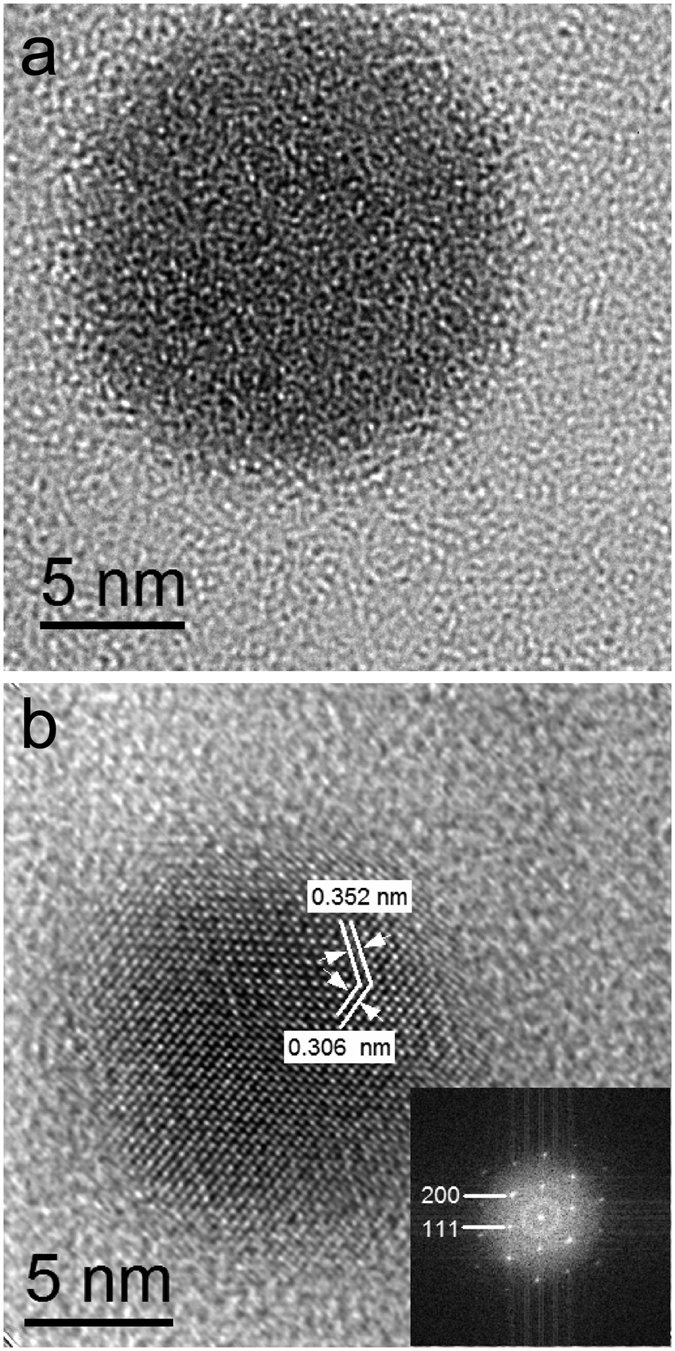
High resolution transmission electron microscopy (HRTEM) images of the amorphous (**a**) and crystalline (**b**) Ge_2_Sb_2_Te_5_ nanoparticles (GST NPs). (**b**) shows that GST nanoparticle possesses a rock-salt structure, and that imaging is recorded along the <110] zone axis. The lattice parameter given by this HRTEM image is 0.612 ± 0.005 nm, 2% extended compared to the bulk GST. Inset in (**b**) displays the fast Fourier transform of this image with two reflections indicated.

In order to characterize the stoichiometry of the nanoparticles (NPs), energy dispersive X-ray spectrometry (EDX) has been performed. Results show for a large number of particles an average ratio of Ge:Sb:Te = 20:23:57 (±1) at.% for all the samples we produced, demonstrating an extraordinary agreement with the nominal composition of Ge_2_Sb_2_Te_5_ (Ge:Sb:Te = 22:22:56 at.%). An EDX spectrum is shown as an example in Figure S1 of the supplementary information (SI). It is important to control the phase state for the as-deposited NPs, both out of scientific interest but also because of the challenge to amorphize the NPs directly upon deposition allowing subsequent crystallization to be studied. By tuning the deposition settings, both amorphous and crystalline NPs have been successfully produced. The phase state of these NPs was confirmed by high resolution transmission electron microscopy (HRTEM), as shown in Fig. 1. Figure 1a displays the amorphous nature of the as-deposited NPs due to the lack of lattice fringes, which in contrast can be unambiguously observed in crystalline NPs; see Fig. 1b. The measured interplanar spacings are d_111_ = 0.353 nm, d_200_ = 0.306 nm. Associated with the measured value of the angle between these two planes (~54.5°) derived from both the HRTEM image and the corresponding fast Fourier transform (FFT, as shown in inset of Fig. 1b), a rock-salt structure with a lattice parameter of 0.612 ± 0.005 nm is derived. This lattice parameter is about 2% extended compared to the value (0.600–0.601 nm) for bulk Ge_2_Sb_2_Te_5_ PCMs^21^,^22^. We consistently found this 2% extension for a large number of particles and this is also consistent with the observation for GST NPs in another work (0.611 ± 0.002 nm)^23^. The (200) and the (111) planes are labelled in the FFT image (inset in Fig. 1b), suggesting a rock-salt structure with a zone axis of <110].

A core-shell structure is absent for the NPs in the HRTEM images, indicating that the NPs were not significantly oxidized. The FFT image (inset of Fig. 1b) also confirms this because no splitting of diffraction spots can be observed, demonstrating only one kind of crystal structure in the nanoparticle. The HRTEM image also shows the single crystal nature of the nanoparticle. Actually from all the HRTEM images recorded no sign of polycrystalline structures has been observed for the GST NPs, most probably due to the small size of the NPs (less than 20 nm in diameter).

The only difference in directly creating either amorphous or crystalline NPs is the discharge current applied to the magnetron sputtering nanocluster deposition system, leading to a difference in power supplied to the system. In comparison to the crystalline sample, a lower current was used for the amorphous sample (0.30 versus 0.15A). In a previous work, it has been demonstrated that the solubility and the diffusivity of the atoms in the NPs can be tuned by adjusting the deposition settings (i.e., the power and the argon flow), where higher input power indeed caused phase segregation in Mo-Cu NPs^24^. The higher power input leads to a higher kinetic energy to the atoms in the plasma, resulting in the formation of liquid phase clusters. As is well known, an extremely high quenching rate (above 10^9^ K s^−1^ 25,26,27) is required to produce amorphous phase PCMs since they have to be in general poor glass formers. Since the quenching rate is not high enough in the aggregation chamber to form amorphous phase, crystalline NPs develop. When a lower discharge current is used, clusters remain below the melting temperature and atoms randomly coalesce and then form NPs. The energy of the atoms (Ge/Sb/Te) gained in the plasma is sufficient to form a nanoparticle, but not enough to overcome the energy barrier of forming a crystalline structure, where amorphous phase is formed analogous to sputtered amorphous films. This hypothesis can be further confirmed by the fact that the ratio of crystalline NPs among all the NPs produced becomes smaller when helium gas (while the other settings remain the same) is used to accelerate the cooling rate in the aggregation chamber. Note that the critical discharge current required to form the liquid state of GST system is much lower than that of the Mo-Cu system (0.55A), because the melting temperature of Mo-Cu is much higher than the one of GeSbTe.

### Morphology and size distribution

**Figure 2:**
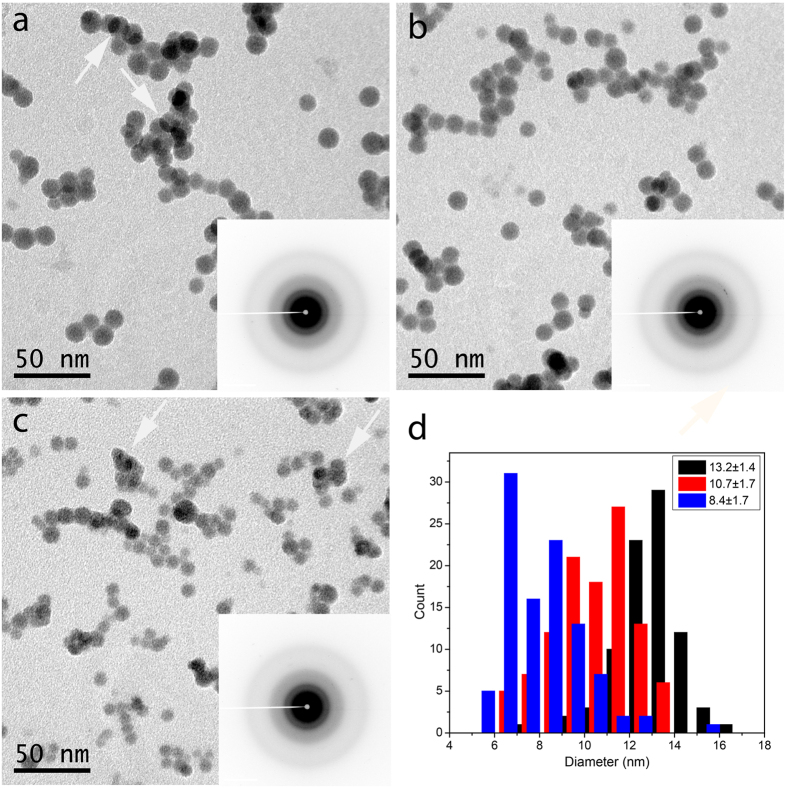
Bright field TEM images of amorphous GST NPs with different sizes. (**a**) large NPs, Ø = 13.2 ± 1.4 nm; (**b**) medium-sized NPs, Ø = 10.7 ± 1.7 nm; (**c**) small NPs, Ø = 8.4 ± 1.7 nm. (**d**) shows the corresponding size distributions for these three samples. The white arrows in (**a**) indicate NP overlap, but not real coalescence, whereas the white arrows in (**c**) indicate NP coalescence.

As described in the previous section, the discharge current has significant impact on the phase state of the as-deposited NPs. So a relatively low discharge current (0.15 A) was used to produce amorphous NPs. The morphology of these NPs was characterized by TEM, as shown in Fig. 2. Figure 2a–c show the GST NPs with different sizes. The amorphous nature of these NPs is confirmed by the selected area electron diffraction (SAED) patterns (insets in the bright field images), owing to the fact that they lack sharp diffraction features, but show a broad halo. The amorphous NPs exhibit approximately spherical morphology, while the crystalline ones display anisotropic features with facets (see Figure S2a). Note that the NPs shown here were produced with hydrogen as extra gas on top of the standard argon gas. No literature has been found to confirm the fact that a potential doping with hydrogen has influence on the crystallization temperature (*T*_*c*_) of PCMs. With more helium as cooling gas in the cluster system, the sizes of the NPs can be significantly reduced due to the two-fold impact of helium: (1) helium is a better thermal conductor than argon, leading to more efficient reduction of kinetic energy of the atoms in the plasma; (2) the presence of helium increases the pressure in the aggregation chamber, resulting in a bigger driving force pushing the NPs out of the aggregation volume to the main chamber^24^,^28^. No helium was used for the preparation of the NPs in Fig. 2a, while 10 and 20 sccm of helium were adopted to reduce the diameter of the NPs shown in Fig. 2b and c, respectively. The average diameters of these 3 samples are 13.2 ± 1.4 nm, 10.7 ± 1.7 nm and 8.4 ± 1.7 nm, as shown in Fig. 2d, which will be referred to as big, medium and small NPs.

All of the NPs in these three samples show a relatively narrow size distribution. Figure 2 also suggests that the size distributions become wider when the average sizes of NPs shrink, i.e., from 10% for the big NPs (13.2 nm) to ~20% for the small ones (8.4 nm); see Table S1 in SI. The as-deposited NPs are well separated when the coverage is low; see individual NPs with low coverage (~7%) in Figure S3 of SI. When the coverage becomes higher (~15% in Fig. 2), which is essential to have enough signal in SAED patterns when performing *in-situ* heating in TEM (see next section), the overlapping between NPs becomes inevitable. However, clear boundaries (see the examples indicated by the white arrows in Fig. 2a) can be observed between NPs, demonstrating that coalescence does not occur in these samples. However, when the amount of helium used is large, a small fraction of coalesced NPs can be observed (2 clusters of coalesced NPs can be seen in area with dimension of 250 × 250 nm^2^), indicated by the white arrows in Fig. 2c. This coalescence most probably stems from the higher purity of the helium aggregation gas than argon. The small NPs always have the tendency to coalesce in order to reduce their surface energy, making coalescence energetically favorable. The impurities on the surface of the NPs, generally in the form of a thin oxide shell, play a role in preventing the NPs from coalescing. In our experiment, helium has a higher purity than argon (99.9999% vs 99.999%). When only argon is used, the small amount of impurity causes an oxide shell (rich in Ge, because this is the element with the highest oxygen affinity) outside the NPs, that prevents the NPs from coalescence. When more pure helium gas is used, less oxide will be formed and therefore coalescence can take place. Moreover, when helium is used the NP size is reduced and thereby the driving force for coalescence is increased, as shown in Fig. 2c.

### Size-dependent crystallization

**Figure 3:**
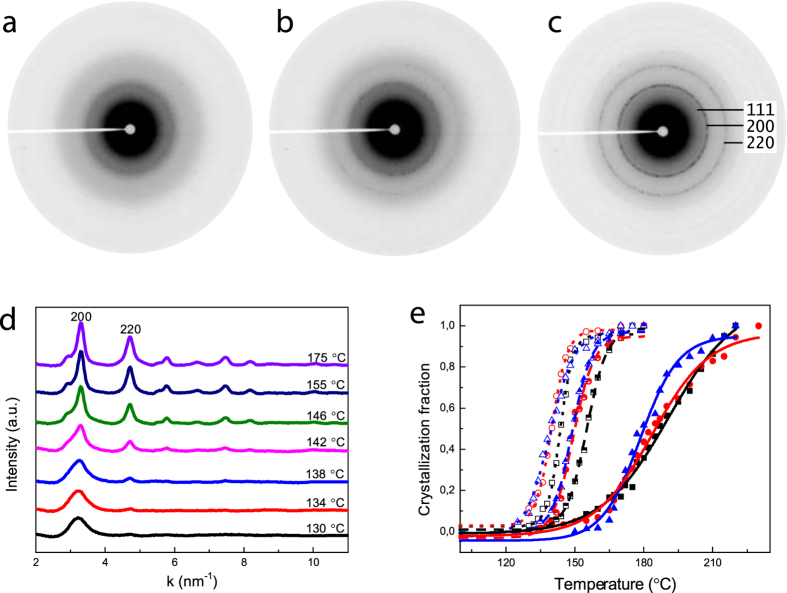
Crystallization of GST NPs via *in-situ* heating. (**a**–**c**) show the selected area electron diffraction patterns of the big NPs shown in Fig. 2a at room temperature, 140 and 175 °C, respectively. Only an amorphous halo exists in the diffraction pattern recorded at room temperature. Some faint, discrete diffraction spots appear when the sample is heated to 140 °C, and finally sharp diffraction rings consisting of discrete spots confirm the phase transition process. (**d**) Evolution of the diffraction patterns as a function of the heating temperature. (**e**) The normalized phase transformation fraction as function of temperature for three different sets of samples, where each set comprises three samples with different average NP diameters. The open symbols are for the NPs produced with hydrogen, while the half open and solid symbols are for the NPs produced with low and high amounts of methane, respectively. Black, red and blue colour means big, medium-sized and small NPs in each session, respectively. The continuous curves in this figure are the fitting results using the Boltzmann function.

Crystallization is one of the most relevant properties to be explored when PCMs are scaled down as it also directly links to the stability of the amorphous phase. Because of the small scale (~10 nm) of the objects and therefore extremely low mass (in nanogram scale), it is extremely difficult to study the crystallization of the NPs by for instance conventional differential scanning calorimetry (DSC). *In-situ* heating in a TEM therefore has been performed to characterize the crystallization process for the GST NPs. As can be observed in Fig. 3a–c, the broad amorphous halo of the GST NPs changes into sharp rings in the diffraction patterns under heating. The evolution of the diffraction patterns as a function of temperature during *in-situ* heating in TEM indicates the crystallization process of GST NPs, as shown in Fig. 3d. By fitting the evolution of the intensity for {220} planes (as shown in Fig. 3e), the *T*_*c*_ of the GST NPs have been derived (details of the methods, see section 2 of the SI).

The *T*_*c*_ derived from fitting are 143.8 ± 0.5 °C, 139.8 ± 0.3 °C and 138.3 ± 0.6 °C for the big, medium and small NPs. Note that the *T*_*c*_ for bulk GST range from 150 to 160 °C for different measurements^29^,^30^,^31^,^32^,^33^. It is noticeable that the coalescence of the NPs produced with 20 sccm helium flow has negligible impact on the *T*_*c*_ of the NPs due to the small number of coalesced NPs compared to the total number of NPs (2 clusters vs ~100 NPs in Fig. 2c). Hence, a (weak) size-dependence of crystallization is revealed here for GST NPs. Interestingly the *T*_*c*_ decrease when the average sizes of nanoparticles reduce, which is opposite to the observation for GeTe NPs^12^,^13^. 1^st^ order derivatives of these fitting curves have been obtained and are depicted in Figure S4. It is noticeable that the full width at half maximum (FWHM) (of the peaks in the derivative curves) for the small NPs is slightly larger than that of the big NPs, indicating that a wider temperature range is needed to completely crystallize the small NPs; see Table S1 in SI.

Furthermore, the bright field TEM images for NPs after heating demonstrate that the GST NPs do not evaporate or coalesce during heating, as shown in Figure S5 in SI. The only visible alteration from these images is the more apparent electron scattering contrast, as the crystalline part usually appears darker in bright field image. Even at the overlapping boundaries, the circular rim of these NPs remains very clear. In contrast, significant sintering of NPs was observed in GeTe NPs by chemical synthesis during crystallization^13^.

### Enhancement of the amorphous stability via methane

As shown in the previous section, the *T*_*c*_ of the GST NPs slightly decreases when the average sizes of the NPs reduce. Although the decrease here is not dramatic, further down-scaling (e.g. to sub 5 nm) is likely to create some challenges for the stability of the amorphous phase. However, a facile method to increase the *T*_*c*_ (therefore the stability of the amorphous phase) of the GST NPs has been discovered. Instead of using hydrogen as extra gas, methane was used to initiate the deposition process. NPs with different sizes have been produced both with low and high amounts of methane, as shown in Figures S6 and S7 in SI. Figure S6 depicts the morphology of the GST NPs produced with a low amount of methane. Helium was also employed here to reduce the sizes of the NPs. The average diameters of these three samples (Figure S6a–c) are 15.1 ± 1.4 nm, 9.8 ± 1.9 nm and 7.9 ± 1.6 nm, respectively. Sizes distributions of these 3 samples are shown in Figure S6d, displaying a narrow size dispersion of these samples. A high amount of methane was further used to confirm that the incorporation of methane plays a role in stabilizing the amorphous state of the GST NPs. The TEM images in Figure S7 depict the morphology of these NPs. The average diameters of these three samples (Figure S7a–c) are 16.8 ± 1.4 nm, 14.5 ± 1.6 nm and 10.7 ± 1.8 nm. Narrow size distributions of these 3 samples are displayed in Figure S7d. Similar to the NPs produced with hydrogen, the smaller the NPs are, the wider the size distribution of the NPs become. Coalesced NPs cannot be observed in the bright field TEM images, but only the overlapping of NPs with distinct boundaries. The spherical shape of the NPs indicates their amorphous nature, which is also confirmed by the SAED patterns (insets in Figures S6 and S7).

As shown in the SI (Figure S8 and section 3), we could determine by EDS that the NPs produced with the addition of a high amount of methane contained significantly more carbon than the NPs produced with hydrogen (when only the four elements C, Ge, Sb and Te are considered we found 22 ± 11 at.% C and 8 ± 2 at.% C, respectively, whereas a negligible amount of carbon is found for the silicon nitride membrane substrate as reference). The detected amount of carbon can be present both within and surrounding the NPs. Moreover, accurate quantification of the carbon concentration with EDS, particularly for large surface area material such as based on NPs, is by itself already difficult. Therefore, we use the qualitative description of low and high amounts of methane in the present work, where our results still demonstrate that the carbon concentration connected to the NPs increase when going from the NPs produced with hydrogen to ones with a low amount of methane and then to ones with the high amount of methane.

Similar to the previous section, *in-situ* heating in TEM was performed to characterize the crystallization of these NPs produced with the addition of methane. The transformed phase fractions as function of temperature for these NPs are depicted in Fig. 3e. The half open symbols are data for the NPs produced with a low amount of methane, while the solid symbols in this figure denote the data for the NPs produced with high amount of methane. The black, red and blue colours represent the big, medium and small NPs, respectively. The *T*_*c*_ obtained through fitting are 155.2 ± 0.2 °C, 149.2 ± 0.5 °C, 148.8 ± 0.2 °C for the big, medium and small NPs produced with low amount of methane. In comparison to the *T*_*c*_ derived for the NPs produced with hydrogen, a significant increase (~10 °C) in *T*_*c*_ is obtained. Furthermore, the high amount of methane during production leads to even higher *T*_*c*_, i.e., 190.8 ± 1.7 °C, 183.7 ± 1.1 °C, and 177.9 ± 1 °C for the big, medium and the small NPs, respectively. Hence a strong effect (more than 35 °C increase) of methane on the *T*_*c*_ has been observed here for the GST NPs.

Figure S4 presents the 1^st^ order derivatives of the fitting curves in Fig. 3e, where the *T*_*c*_ of these samples was derived from the peak temperature. From this figure, it is noticeable that the incorporation of methane not only influences the onset temperature of crystallization, but also the crystallization speed. In comparison with the ones produced with hydrogen (open symbols in Fig. 3e) and low amount of methane (half open symbols in Fig. 3e), the FWHM for the ones with high amount of methane (solid symbols in Fig. 3e) is significantly larger, suggesting that a larger temperature range is required to accomplish the crystallization process.

It is well-known that crystallization involves two processes: nucleation and subsequent crystal growth. The width in temperature (non-abruptness) of the crystallization process originates from the difference in incubation time for nucleation of the various NPs, because the crystal growth rate is fast (above 10^−6^ m s^−1^ for GST thin films at this temperature)^34^ once nucleation has occurred in an NP and will finish within the time for stabilizing the specimen (30 s). The overall crystallization curves shown in Fig. 3e therefore represent the ensemble of the crystallization of individual NPs with their variation in the incubation times for nucleation. For the temperature interval we consider the nucleation rate increases with time and temperature. Therefore, we would expect a more abrupt crystallization when this transition occurs at higher temperatures. However, in the case of methane addition, where the transition is shifted to higher temperature, we observe a slower transition. This thus implies that the addition of a high amount of methane retards the overall process of crystallization significantly. The effect of methane is thereby similar to carbon doping which retards the crystallization process in Ge_2_Sb_2_Te_5_ films^35^.

**Figure 4:**
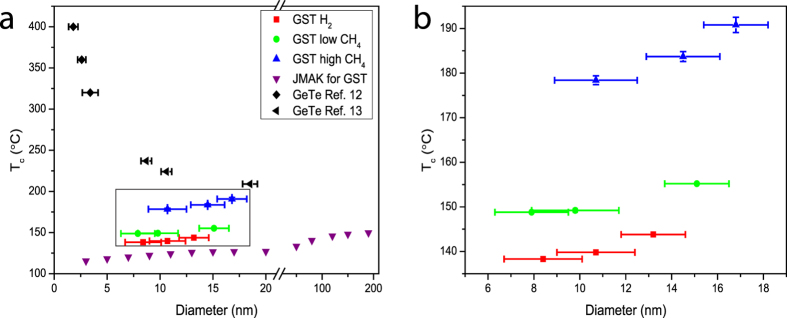
(**a**) Size-dependence of the crystallization temperatures for GST and GeTe NPs. The blue triangles, green circles and red squares are the *T*_*c*_ data for GST NPs at different diameters and different incorporation gases as determined in the present work. The data in black are *T*_*c*_ for GeTe NPs at different diameters as taken from literature^12^,^13^. Opposite size-dependence of *T*_*c*_ can be observed for GeTe and GST NPs. Numerically calculated data based on experimental data from GST nanowires are displayed in purple triangles^15^, demonstrating the same trend as the GST NPs in the present work. (**b**) The close-up of the rectangular area in (**a**).

The *T*_*c*_ as a function of size and the incorporated methane is illustrated in Fig. 4. In addition, the *T*_*c*_ of GeTe NPs (black diamonds and triangles) are also shown in this figure for contrast^12^,^13^. All the values of the average diameters and the *T*_*c*_ are displayed in Table S2 of the SI. Similar size-dependent trends are observed for all of these samples, no matter the NPs are produced with hydrogen or methane. In comparison to the size-dependence of the *T*_*c*_, the methane gas produces a much more pronounced effect.

Size-dependent crystallization has been reported in many glass forming liquids. For example, lower crystallization temperature has been reported when the sizes of Si NPs reduce^36^. However, the weak relation between the size and *T*_*c*_ is unexpected for GST NPs. In previous works^10^,^15^, the activation energy for crystal growth has been reported to reduce from 2.34 eV to 1.86 eV for Ge_2_Sb_2_Te_5_ nanowires when their widths reduce from 190 nm to 20 nm. Meanwhile, the nucleation rates increase at least 4 orders of magnitudes due to surface-induced heterogeneous nucleation. Based on these two factors, it is understood that the GST NPs show decreasing *T*_*c*_ when the average sizes decline.

Analogous to previous work^37^, we performed numerical calculations based on Johnson-Mehl-Avrami-Kolmogorov (JMAK) theory and adopting the same heating rate as used in the *in-situ* TEM measurements in order to simulate the crystallization curves for the relevant sizes around those of the NPs, (for details, see section 4 of SI). Utilizing data for size-dependent activation energy and nucleation rate originally found for GST nanowires^15^, the simulated size-dependence of crystallization for GST NPs is presented in Fig. 4 (purple triangles). The *T*_*c*_ of NPs with a diameter of 200 nm in these numerical calculations is set to ~150 °C, close to that of bulk GST. The same trend of size-dependence can be readily observed for the modelling and the experimental data we obtained for GST NPs (red, green and blue points), i.e., a slight decrease in *T*_*c*_ when the sizes become smaller. However, the *T*_*c*_ observed for the non-doped GST NPs (red squares in Fig. 4) in the present work are roughly 20 °C higher than the theoretically predicted values, suggesting that the size-dependence of crystallization for the current GST NPs is weaker than that of nanowires.

In a previous work^23^, a higher *T*_*c*_ (~180 °C) for GST NPs with a diameter of 5.7 ± 1 nm was observed compared to that of bulk GST. Nevertheless, this difference in *T*_*c*_ probably originates from the discrepancy in stoichiometry, as it was also reported that the composition for the NPs (Ge:Sb:Te = 28:27:45) differed considerably from the nominal stoichiometry of Ge_2_Sb_2_Te_5_ (Ge:Sb:Te = 22:22:56). It should be noted that the composition affect the *T*_*c*_ of GST ternary alloy pronouncedly^38^. For instance, the *T*_*c*_ of Ge_2_Sb_2_Te_4_ film is reported as 175 °C^22^. Moreover, the alumina layer used to cap the NPs in the previous work is also likely to increase *T*_*c*_. Providing a compressive stress to the NPs by the capping layer, the amorphous phase can be stabilized, therefore the transformation from amorphous to crystalline is retarded^39^,^40^,^41^,^42^. No further information on crystallization of GST NPs with varying sizes has been reported by this group. In comparison, the composition (Ge:Sb:Te = 20:24:56, ±1) obtained from the EDS for the NPs we produced here is in remarkably good agreement with the nominal stoichiometry of Ge_2_Sb_2_Te_5_ and no capping layer is used in our work.

Surprisingly, the opposite size-dependence trend was found for GeTe NPs studied previously (the black data points in Fig. 4a)^12^,^13^, where a sharp increase in *T*_*c*_ was observed for decreasing particle size. GeTe NPs with a diameter of 1.8 nm exhibit a *T*_*c*_ of 400 °C in comparison to ~180 °C for bulk GeTe.

The minor size-dependence in the crystallization temperature of the GST NPs observed here is favourable for memory applications, because simultaneously, it is expected that the melting temperatures (*T*_*m*_) will drop as the NPs become smaller. A sharp decrease in *T*_*m*_ with decreasing size has been widely observed in many different systems, like Au NPs^43^, Ag NPs^44^, and Sn NPs^45^. Since the SET (crystallization) process is usually operated at temperatures between *T*_*c*_ and *T*_*m*_, a strongly increasing *T*_*c*_, as observed for GeTe, would be problematic, because it generates a smaller operation window for crystallization and an accompanying reduction in maximum crystallization rate.

Enhancing the stability of amorphous phase in a proper range is usually favourable since higher *T*_*c*_ represents better data retention (at operating temperature e.g. up to 100 °C). The present work demonstrates that the extra gas (methane) plays a promising role to stabilize the amorphous phase of the GST NPs. It has been demonstrated that it is possible to form carbon as by-product when methane is used to accelerate the nucleation in the cluster system^28^. Therefore, carbon can be randomly doped into the NPs during the nucleation of the NPs inside the cluster source. Note that a carbon shell was unambiguously detected for Cu NPs in HRTEM image in a previous work by our group^28^, yet it cannot be clearly observed for the GST NPs in HRTEM image, as shown in Figure S9a of the SI. Considering (1) the similarity of the method to produce NPs here and the magnetron sputtering utilized to produce carbon doped GST thin films and (2) the strong effect of methane addition on *T*_*c*_, we draw the conclusion that this increase of *T*_*c*_ is mainly due to carbon doping and not a carbon shell. In GST and GeTe films, carbon doping has been reported as an effective method to stabilize the amorphous phase. For example, 9 at.% of carbon dopant in Ge_2_Sb_2_Te_5_ films results in an increment of ~10 °C of *T*_*c*_ compared to the non-doped film. Further, a high amount of doping (18 at.% of carbon) leads to an increment of 40 °C of *T*_*c*_^35^. This influence of doping on NPs is surprisingly prominent since the partial pressure of methane is very low (less than 1% even with the high amount of methane) compared to the deposition pressure (determined mostly by argon and helium flow and the target atoms) during sample preparation. In GeTe films, the carbon dopant influence the *T*_*c*_ more intensely, where 4% of carbon dopant results in a *T*_*c*_ of ~290 °C compared to ~180 °C for the non-doped GeTe film^46^. In both cases, the activation energy for crystallization have been increased pronouncedly by carbon doping. To conclude, the tuneable *T*_*c*_ of the GST NPs using methane as an incorporation gas provides an attractive way to enhance the amorphous phase stability. As described above, carbon doping retards the amorphous to crystallization transition, leading to a reduction in crystallization speed around and below *T*_*c*_. However, the activation energy for crystallization for carbon doped GST films is higher than that of the non-doped ones^35^. Therefore, the crystallization speed at real operation temperature, which can be 400–500 °C, can probably reach or exceed the speed of non-doped GST. This is very favourable, because then both the data retention as well as SET speed are improved.

### Crystallography of the *in-situ* heated GST NPs

The crystal structure after crystallization was determined by HRTEM for the NPs produced with hydrogen and the low amount of methane. Despite some visible {220} facets in the HRTEM image (on the left and right sides of the NP) shown in Figure S9a, the faceting is hardly visible in overview images (with lower magnification). A difference between the crystalline NPs directly formed in our NPs deposition system and the ones formed by heating initial amorphous NPs can be expected, because the former ones form directly out of liquid NPs, whereas the latter form inside initially glass-like NPs. This for instance has a clear impact on the temperature and viscosity of the NPs material in which the crystals nucleate and grow. For the crystals forming in the liquid NPs the temperature is higher and the viscosity lower giving much more flexibility during crystallization to affect the initial spherical shape of the NPs to facetted ones. On the other hand, for the crystals forming in the glass-like NPs the temperature is lower and the viscosity higher giving much more rigidity during crystallization not allowing clear overall shape-changes of the initial spherical shape of the NPs to facetted ones.

Surprisingly, a mixture of metastable rock-salt and rhombohedral structures is observed. For the rock-salt structure, the lattice parameter obtained from HRTEM images is consistent with the one of the as-deposited crystalline NPs, with lattice parameter a = 0.612 ± 0.005 nm, as shown nicely in Figure S9a, SI. From Figure S9b, clear vacancy layers (VL) can be observed in the HRTEM image. This VL is normally absent in rock-salt structure due to the random distribution of the vacancies in this structure, indicating that the transition to the more stable rhombohedral structure, which for bulk GST is expected for clearly higher temperatures^26^,^30^,^47^, is already initiated after heating to 175 °C. Moreover, oxide shells or other crystal structures are not observed from these images, suggesting that oxidation is insignificant during heating.

The incorporation gas should not play a role for the merging of VL as the mixture of these two structures is observed in the NPs produced with both hydrogen and methane. A possible reason for this is that the transition temperature from rock-salt structure to rhombohedral structure (*T*_*c2*_) is significantly reduced with scaling down of the PCMs. For Ge_2_Sb_2_Te_5_ phase-change films, this temperature has been reported ranging from 240 °C to 370 °C for different measurements^26^,^30^,^47^. However, a size-dependence of *T*_*c2*_ has been observed in Ge_2_Sb_2_Te_5_ films. While the transition temperature from amorphous to rock-salt structure is hardly changed, the *T*_*c2*_ is pronouncedly reduced when the thicknesses of films are reduced. At a thickness of 20 nm, the later temperature has been lowered to ~200 °C^48^. Since the diameters of the GST NPs in the present manuscript are smaller than 20 nm, an even lower temperature is possible for the transition from rock-salt to the trigonal structure. The samples we performed HRTEM characterization on have been heated to 175 °C, so probably we have reached the onset temperature of the second phase transition. However, this transition is not detected in the SAED patterns in TEM. Further systematic work is needed to understand the mixed structure.

## Conclusions

We have introduced a facile method to produce phase-change Ge_2_Sb_2_Te_5_ (GST) nanoparticles (NPs) with excellent crystallinity, size and composition control. The rock-salt structure of the GST NPs exhibit ~2% extension in lattice parameter compared to bulk GST. *In-situ* heating of amorphous NPs in transmission electron microscope shows that crystallization is size-dependent, where the crystallization temperatures (*T*_*c*_) decrease when the NP sizes decrease. However the difference in *T*_*c*_ is only a few Kelvin when the sizes of NPs are typically reduced by a factor of 2 (from 17 to 8 nm), which is preferable for applications. Numerical modelling via JMAK theory illustrates that surface-induced heterogeneous nucleation is able to explain this size dependent crystallization. The presence of methane gas during deposition gives rise to a large increase in *T*_*c*_ of ~35 °C compared to when methane is absent and hydrogen is used instead. This work shows for the first time (i) the size-dependent crystallization of GST NPs and (ii) the tuneable crystallization temperatures of GST NPs when methane gas is added during sputtering. Hence, our approach is relevant for the design of phase-change memories and PCMs based devices with an active PCM size clearly smaller than 20 nm, which is very hard to achieve with optical lithography.

## Experimental Methods

### Ge_2_Sb_2_Te_5_ nanoparticles preparation

The Ge_2_Sb_2_Te_5_ (GST) nanoparticles (NPs) with different phase states and sizes were produced by magnetron sputtering with inert gas condensation in a home-modified nanoparticle system Nanosys50 from Mantis Deposition Ltd. The sample chamber was evacuated to a base pressure of 10^−8^ mbar. Argon (purity of 99.9999%) was used to produce the supersaturated vapour via magnetron sputtering the Ge_2_Sb_2_Te_5_ target (purity of 99.99%). Hydrogen or methane was used to facilitate the formation of clusters. Note that the amount of hydrogen or methane used can only be specified qualitatively, because a gauge measuring the gas flow precisely is lacking. Different discharge currents were used to modify the as-deposited phase of the NPs, i.e., 0.15 A for amorphous NPs and 0.3 A for crystalline NPs. Helium (purity of 99.999%) was used to tune the sizes of the NPs. The NPs were subsequently deposited on the (holey/continuous) carbon support films (on Cu grids) in the main chamber.

### Ge_2_Sb_2_Te_5_ nanoparticles characterization

The morphology of the NPs was characterized instantly after deposition by transmission electron microscope (TEM, JEOL 2010) at 200 kV. The crystalline structure was characterized by high-resolution TEM in JEOL 2010 F at 200 kV. The composition of the NPs was characterized by energy disperse X-ray spectrometry (EDS) attached to the TEMs (Thermo Instruments on the JEOL 2010 and Bruker Quantax on the JEOL 2010 F). The *in-situ* heating in TEM (JEOL 2010) was performed to determine the crystallization process right after the deposition of the GST NPs. Single tilt heating holder (Gatan Model 628) with the temperature controlled by a SmartSet Hot Stage controller (Gatan Model 901) was used for heating. The temperature accuracy of the indicator is about 0.1 °C. Although the absolute error to determine the actual temperature of the observed TEM specimen area can be substantially larger it is essential to note here that the observed *differences* in temperature (e.g. as a function of particle size or methane addition) when reproducing experiments very carefully can indeed be very small. Heating rates adopted here were about 1 °C min^−1^ at temperatures above 100 °C. Selected area electron diffraction patterns were recorded at the same area when the sample was heated to a certain temperature (from room temperature to 175–230 °C for different samples). The area selected to record the diffraction patterns are close to the copper bar at the edge of the whole TEM membrane, to minimize the temperature gradient between the heating area and the NPs. During heating, the electron beam was shifted to the copper bar in order to avoid the influence of the electron beam on the crystallization of the NPs^47^. At each temperature step (2 °C), a time interval of 30 seconds was taken for the sake of stabilization of the TEM membrane to avoid the influence of drifting caused by thermal expansion. In the experiments we adopted the largest selected area diffraction aperture (JEOL 2010) for all the measurements, which probes an area of the sample with a diameter of about 2.5 μm. Since the NP density on the sample is in the range from 1500–3000 μm^−2^, the number of particles analyzed to determine a *T*_*c*_ is typically in-between 7500–15000. The azimuthal integration of the diffraction patterns was performed by the PASAD plug-in (http://www.univie.ac.at/pasad/) in Digital Micrograph software in order to derive the evolution of the diffraction intensity with temperature^49^. The errors in *T*_*c*_ have been determined as the standard error directly obtained from our fitting procedure in the software (Origin 8.5). The fitting quality was estimated by the adjusted R-squares, which are in the range from 0.988 to 0.998 for all the curves in Fig. 3e of the main text.

## Additional Information

**How to cite this article**: Chen, B. *et al*. Size-dependent and tunable crystallization of GesbTe phase-change nanoparticles. *Sci. Rep.* **6**, 39546; doi: 10.1038/srep39546 (2016).

**Publisher's note:** Springer Nature remains neutral with regard to jurisdictional claims in published maps and institutional affiliations.

## Supplementary Material

Supplementary Information
