# Supplementary material for: Size-dependent and tunable crystallization of GeSbTe phase-change nanoparticles
Source: Sci Rep. 2016 Dec 20;6:39546. doi: 10.1038/srep39546 (PMC5172365; doi:10.1038/srep39546)
Supplement: Supplementary Information [file srep39546-s1.pdf]

Supplementary information for

Size-Dependent and Tunable Crystallization of GeSbTe Phase-Change Nanoparticles

Bin Chen, Gert H. ten Brink, George Palasantzas, Bart J. Kooi[*]

Zernike Institute for Advanced Materials, University of Groningen,
Nijenborgh 4, 9747AG Groningen, The Netherlands

1. Figures

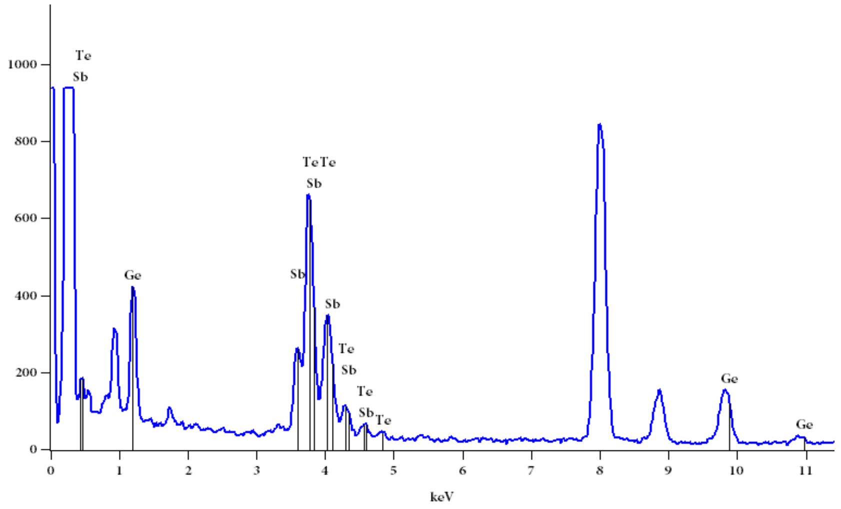

Figure S1. Energy dispersive X-ray spectrum, providing information on the stoichiometry of the NPs. The ratio of Ge:Sb:Te derived from the spectrum is 20:23:57 (±1), showing good agreement with the nominal composition of $Ge_2Sb_2Te_5$.

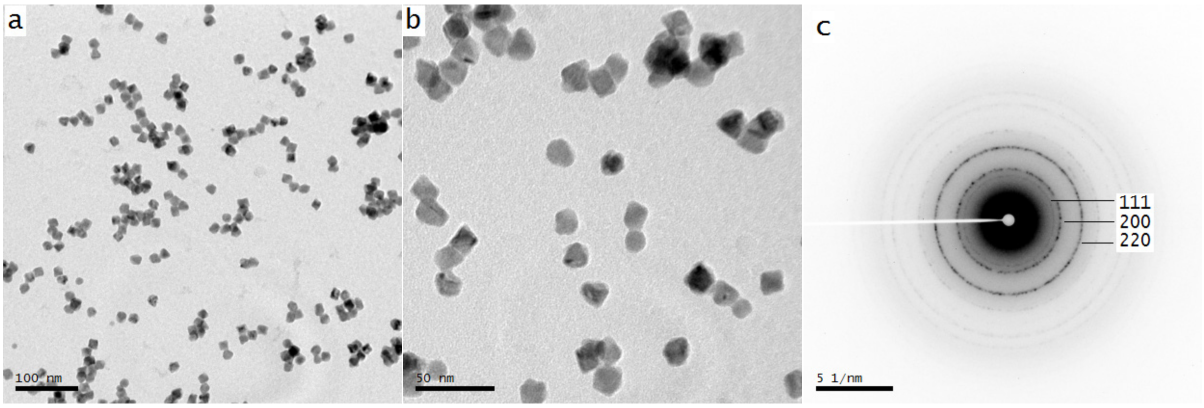

Figure S2. Bright field TEM images (a-b) showing the morphology of the as-deposited crystalline GST NPs. The NPs exhibit anisotropic features with facets, i.e., triangular and rectangular-like shape. (c) Selected area electron diffraction (SAED) pattern of sample shown in a-b. Sharp diffraction spots confirm the crystalline nature of these NPs.

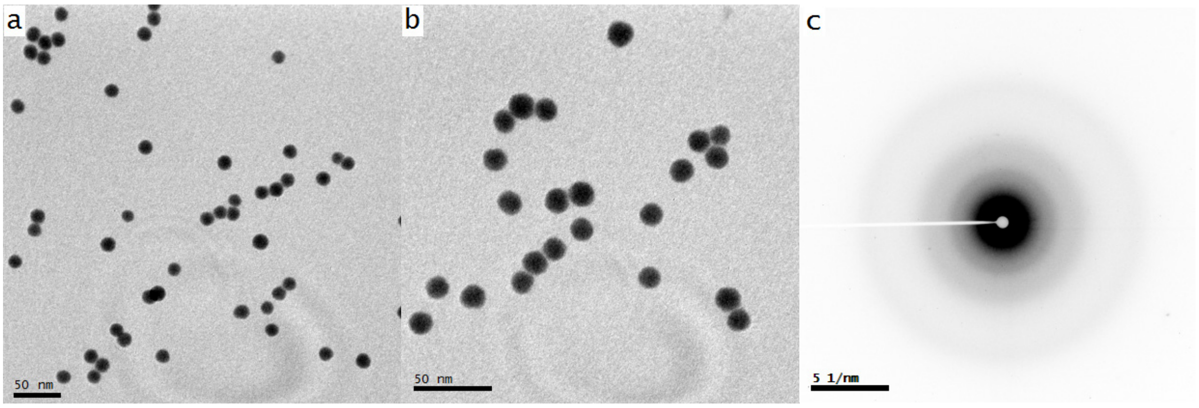

Figure S3. Bright-field TEM images showing a low NP coverage (~7.3 %). Overlapping of NPs is not observed in the images (a) and (b). The lack of Bragg diffraction spots in the electron diffraction patterns (c) demonstrates the amorphous nature of these NPs.

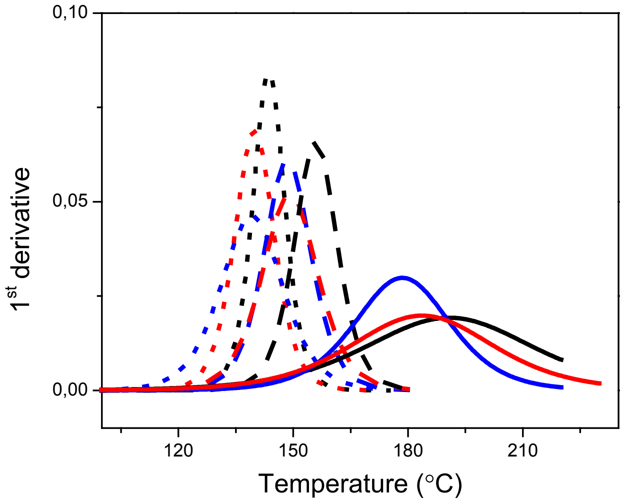

Figure S4. The 1st derivatives of the fitted curves in figure 3e of the main text, with the peak indicating the crystallization temperature (T_c) of each sample. Black, red and blue colour means big NPs, the medium-sized NPs and the small NPs in each session.

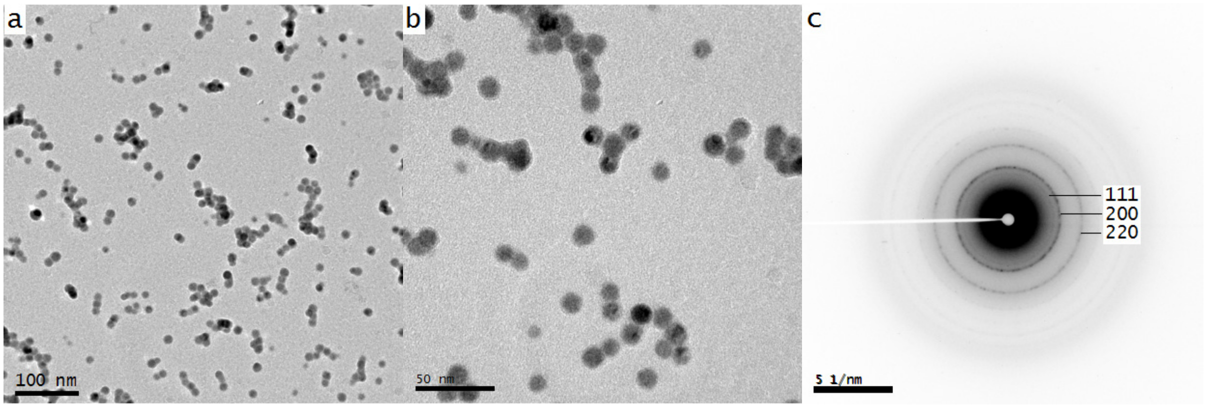

Figure S5. TEM images for GST NPs after heating. Bright field TEM images (a) and (b) show the morphology of NPs after crystallization, indicating that aggregation does not occur. SAED pattern (c) confirms the crystalline nature after crystallization.

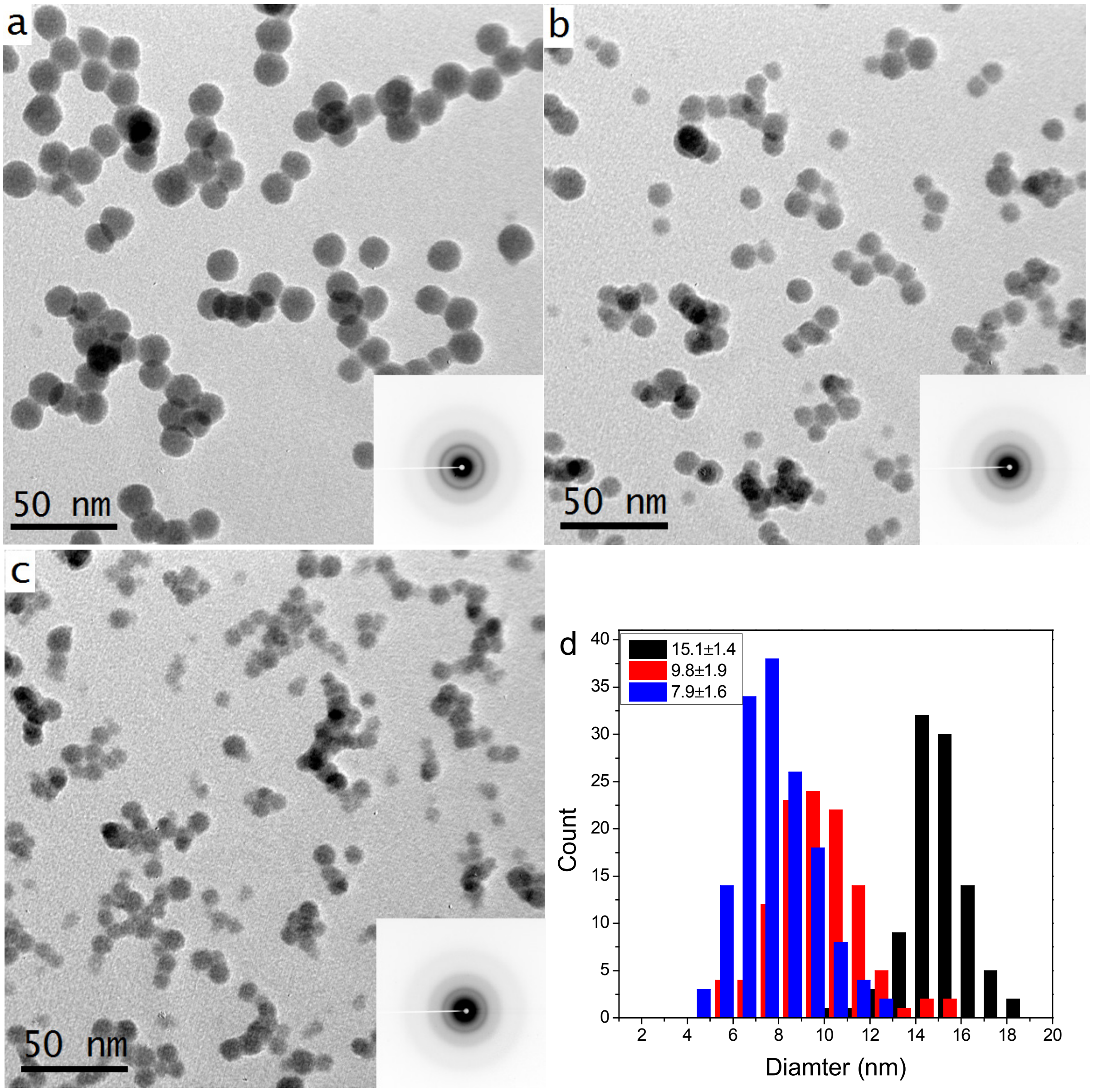

Figure S6. TEM images (a-c) showing the morphology of the GST NPs produced with a low amount of methane. The amount of helium used was 0 (a), 10 (b) and 20 (c) sccm. The average diameters of the NPs shown in the three pannels are 15.1±1.4 nm (a), 9.8±1.9 nm (b) and 7.9±1.6 nm (c). (d) Size distributions of the three different samples shown in a-c. Insets SAED patterns in the images in a-c indicate the amorphous nature of the NPs.

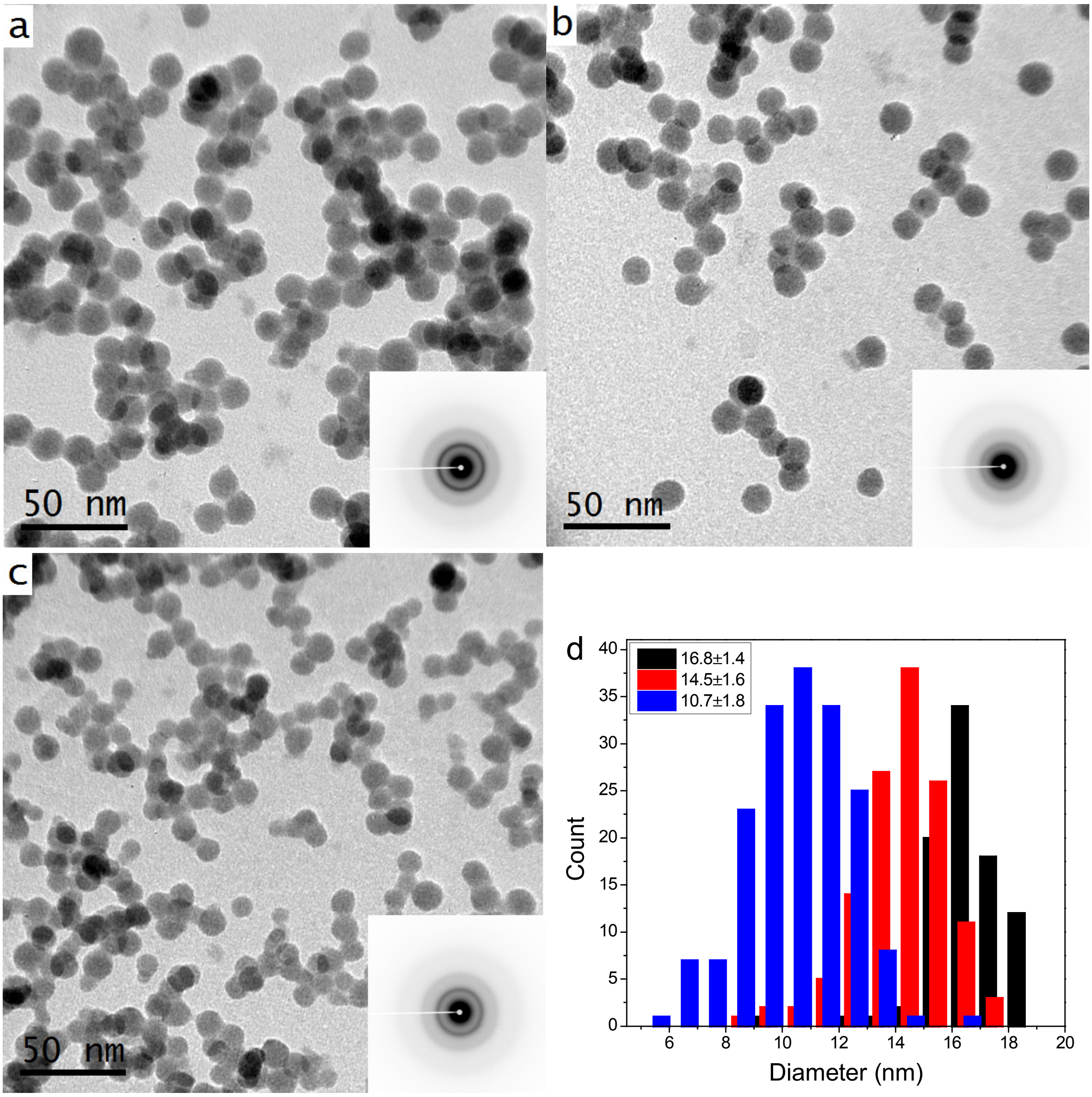

Figure S7. TEM images (a-c) showing the morphology of the GST NPs produced with a high amount of methane. The amount of helium used was 0 (a), 10 (b) and 20 (c) sccm. The average diameters of the NPs shown in the three pannels are 16.8±1.4 nm (a), 14.5±1.4 nm (b) and 10.7±1.8 nm (c). (d) Size distributions of the three different samples shown in a-c. Insets SAED patterns in these images a-c indicate the amorphous nature of the NPs.

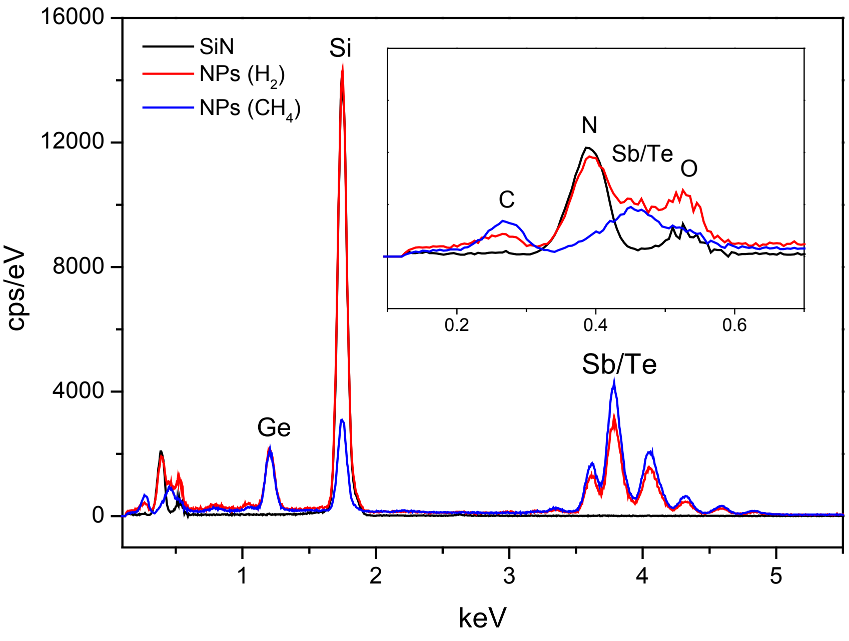

Figure S8. Carbon concentration of the nanoparticles. Carbon concentration was measured for GST NPs produced with H_2 (red curve) and high amount of CH_4 (blue curve), showing a remarkable difference in carbon concentration when the spectra of the two types of GST NPs are normalized compared to the intensity of the Ge L-line and where the spectrum of the silicon nitride membrane reference is normalized to the intensity of the Si K-line measured in case of the GST NPs produced with H_2 (red curve). Inset is the close-up at lower energy, showing the difference in carbon concentration.

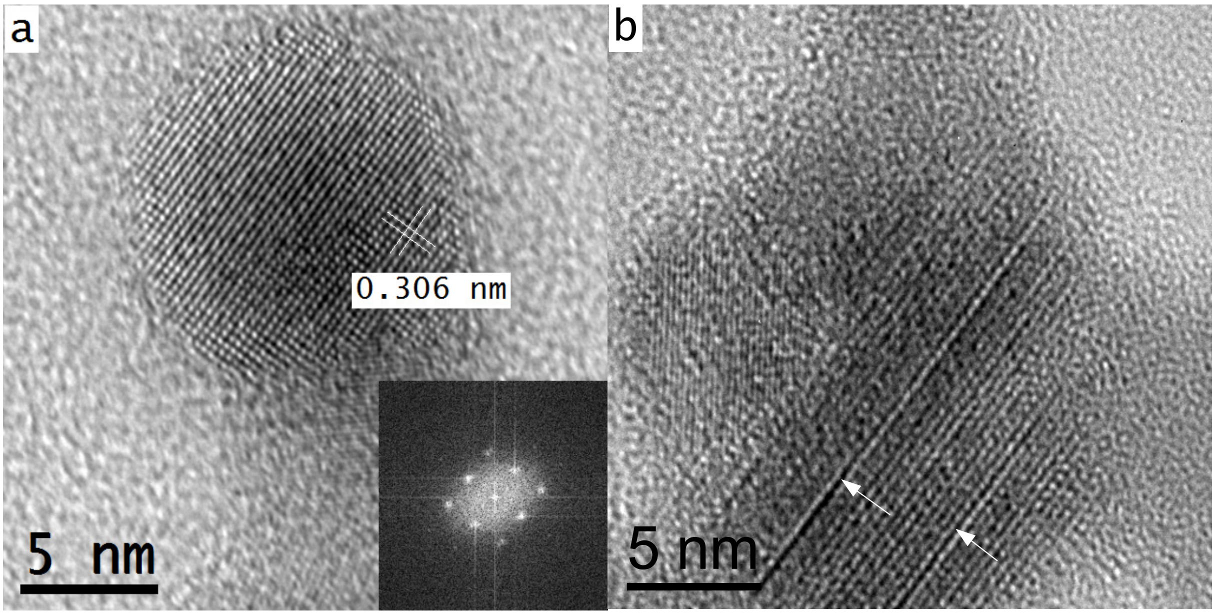

Figure S9. HRTEM images showing the FCC and trigonal (symmetry breaking of FCC by vacancy layers developing on specific (111) planes of FCC) crystal structures of within GST NPs after heating. (a) HRTEM image shows the rock-salt structure in [100> zone axis with interplannar spacing d_{200}=0.306 nm, suggesting consistent lattice parameter to the one of the as-deposited crystalline NPs.

Inset shows the fast Fourier transform of this NP, confirming the rock-salt structure. (b) HRTEM image shows the vacancy layers formed in NPs as indicated by the white arrows. These layers show the onset of the transition from the metastable (rock-salt) to the stable (rhombohedral) phase of GST where initially vacancy layers develop which transform later to Van-der-Waals gaps.

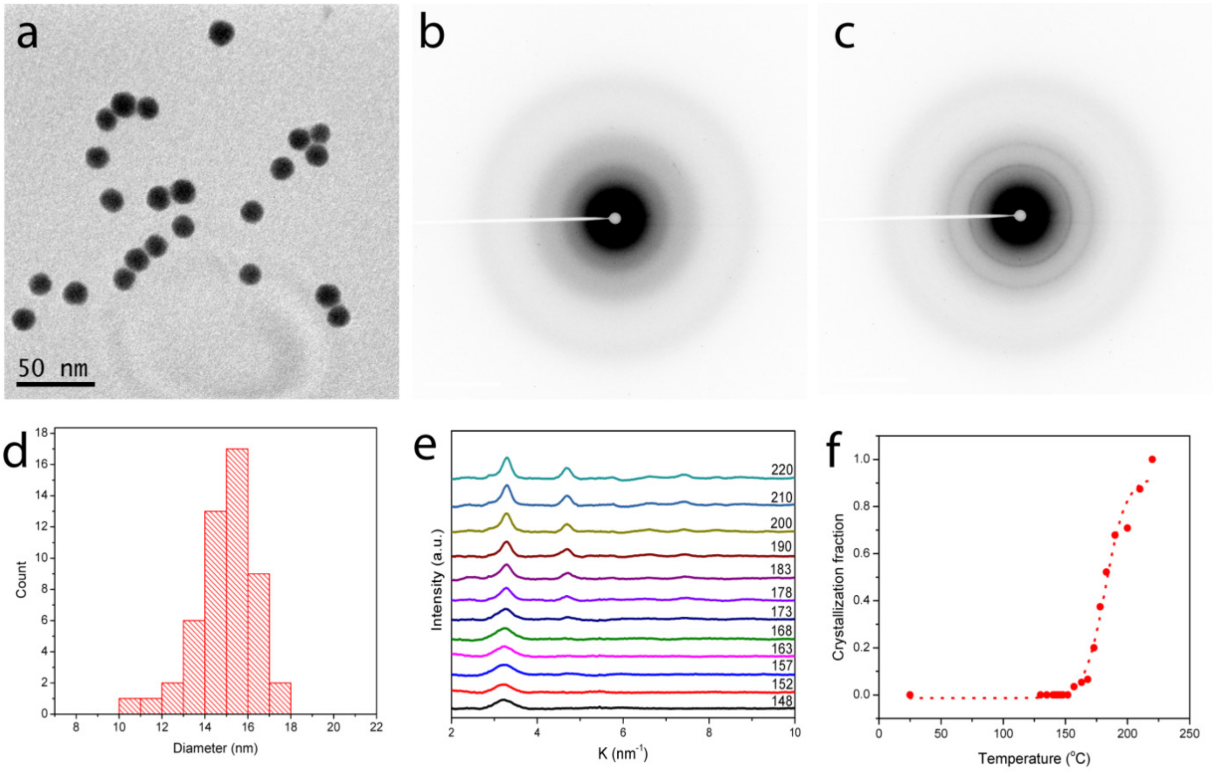

Figure S10. Crystallization of nanoparticles with low coverage (9%). (a) showing the morphology of the as-deposited GeSbTe nanoparticles (NPs) produced with high amount of CH_4. (b) demonstrating the selected area electron diffraction (SAED) patterns at room temperature. The lack of sharp rings indicate the amorphous nature of the NPs. (c) showing the SAED patterns after in-situ the NPs to 220 ^{o}C. The diffraction rings for {200} and {220} planes are evidently observed. (d) demonstrating the narrow size distribution of the NPs, providing an average size of 15.2±1.4 nm. (e) The evolution of integrated intensity of diffraction patterns as a function of temperature. Note SEAD patterns for lower temperatures (130-146 ^{o}C) were recorded, but no signal of crystallization peaks can be observed. Thus only patterns above 148 ^{o}C are depicted in (e) for simplicity. (f) The normalized crystallization fraction derived from (e) as a function of temperature, giving a crystallization temperature 182.3±1.2 ^{o}C for the NPs shown in (a). This crystallization temperature is in very good agreement with the size dependence for the NPs grown with high CH_4 in the main text; see the blue triangles in Figure 4 in the main text. This measurement shows that the crystallization temperature dose not depend detectably on the concentration of NPs.

Table S1. The size dispersion and full-width half maximum (FWHM) of the temperature peaks displayed in figure S4. The FWHM because of the slow continuous heating can also be used as an indication of the crystallization speed.

	**Big**		**Medium**		**Small**	
	FWHM (°C)	Size dispersion (%)	FWHM (°C)	Size dispersion (%)	FWHM (°C)	Size dispersion (%)
**H_2**	9	10.6	11	15.9	17	20.2
**Low CH_4**	13	9.2	17	19.4	15	20.3
**High CH_4**	44	8.3	40	11.3	28	16.8

Table S2. The crystallization temperatures (T_c) of all the different types of NPs samples analyzed in the present work.

	**Big**		**Medium**		**Small**	
	Size (nm)	T_c (°C)	Size (nm)	T_c (°C)	Size (nm)	T_c (°C)
**H_2**	13.2±1.4	143.8±0.5	10.7±1.7	139.8±0.3	8.4±1.7	138.3±0.6
**Low CH_4**	15.1±1.4	155.2±0.2	9.8±1.9	149.2±0.6	7.9±1.6	148.8±0.2
**High CH_4**	16.8±1.4	190.8±1.7	14.5±1.6	183.7±1.1	10.7±1.8	178.4±1

2. Crystallinity determination through in-situ heating TEM

Figure 3a-c in main text shows an example of selected area electron diffraction (SAED) patterns recorded for the NPs shown in Figure 2 in main text during their transformation from the amorphous phase to the crystalline one with increasing temperature. At room temperature, the diffraction pattern shows a broad band without any sharp diffraction spots, confirming the amorphous nature of the NPs. When this sample is heated to 140 °C, some faint, discrete diffraction spots emerge, suggesting that crystallization has started. Continuous heating triggers the increase of both the numbers and the intensity of these diffraction spots, demonstrating the progressing of the phase transformation. Finally, the diffraction rings, consisting out of discrete diffraction spots, are formed at 175 °C. The increase of temperature is halted when no obvious increase of diffraction intensity can be observed during a significant increase of the temperature (5 ℃). Too high temperatures are avoided in order to suppress evaporation of the NPs, which is detrimental to the TEM.

Azimuthal integration has been applied to the diffraction patterns in order to derive in a straightforward manner the intensities for different diffraction rings as function of the distance in reciprocal space to the centre point.[1] The interplanar spacings calculated from SAED patterns after heating are d_{200}=0.3058 nm, d_{220}=0.2114 nm, indicating that the lattice parameter obtained by SAED is in good agreement with the one derived from the HRTEM images (see Figure 1b in the main text).

Repeating the azimuthal integration for all the SAED patterns recorded at different temperatures, the evolution of diffraction intensities with temperature is sequentially obtained, as shown in Figure 3d in the main text. Note that in this figure the background has been subtracted after the azimuthal integration. A broad band at the position corresponding to the {200} planes, which can be explained by the existence of short-range order (local structure of Ge/Sb-Te bondings) in these materials, is generally present in the X-ray diffraction and SAED measurements.[2,3] From Figure 3d, it is evident

that the crystallization starts already at 130 °C, indicated by a visible diffraction intensity for {220} planes. Further heating leads to a continuous increase of the diffraction intensities, until the temperature increasing is halted at 175 °C, because no obvious increase of diffraction intensities is observed anymore in-between 170 °C and 175 °C.

Because of the fact that all the diffraction patterns were recorded at the same area, the evolution of the diffraction intensities with temperature is directly indicative for the progress of crystallization (fraction transformed). In order to quantitatively investigate the crystallization process and subsequently obtain the crystallization temperature (T_c) of these NPs, the diffraction intensities of {220} planes were selected as indicative of the fraction of NPs crystallized. The {200} diffraction intensities are less suitable, because of the interference with the initially present broad amorphous halo around the {200} reflections. The crystallization fractions as a function of temperature for NPs produced with hydrogen are depicted by open symbols in Figure 3e of the main text. The large, medium and small sized NPs (13.2±1.4 nm, 10.7±1.7 nm and 8.4±1.7 nm) are represented by black, red and blue colours, respectively. The Boltzmann function was used to fit the data in order to obtain the T_c, which is denoted as temperature where the maximum transformation speed occurs. Good fits have been obtained for all three curves, with adjusted R^2 as 0.9959, 0.9957 and 0.9937 for the big, medium and small NPs respectively.

3. Measurement of carbon concentration

Figure S8 depicts the spectra measured by energy dispersive X-ray spectroscopy (EDXS) attached to the transmission electron microscope (JEOL 2010F). A significantly higher carbon concentration (22 ± 11 at%) is observed for the NPs produced with high amount of CH_4 compared to the ones (8 ± 2 at%) produced with H_2, while the carbon concentration on the SiN membrane (as reference) is negligible. We also observed that the carbon concentration varies remarkably between different measurements. A possible reason for this is that the NPs can also absorb carbon from the environment during transportation and/or inside the TEM because of their large surface to volume ratio, leading to the difference in carbon concentration. The detectable carbon in the nanoparticles produced with H_2 also indicates that some carbon is absorbed by the NPs, because no carbon is introduced during their deposition process. The low sensitivity of EDXS detectors to carbon strongly attributes to the extreme difficulty to accurately quantify the carbon concentration for NPs. It should be noted that the carbon concentration for the NPs is high (for both types of NPs), we are not able to quantify the relative amounts of carbon that are inside or surrounding the NPs. Nevertheless, the significant difference in the carbon concentration measured for the NPs produced with CH_4 compared to the ones with H_2 strongly supports that the difference in crystallization temperature observed for both particle types must be attributed to the carbon incorporation.

4. Numerical calculation for size-dependence of crystallization temperatures

Analogous to a previous work,[4] JMAK theory was used here to perform the numerical calculation for the size-dependence of T_c. The isochronal heating was divided into small isothermal steps. Here the simulation is performed between 27 °C and 227 °C, while 0.1 °C is used as the step length. The heating rates are 10 °C min^{-1} from 25 °C to 130 °C, and 1 °C min^{-1} from 130 °C to 227 °C to simulate the actual heating process used in experiments.

It has been reported that the down-scaling of $Ge_2Sb_2Te_5$ (GST) nanowires induce a transition towards more heterogeneous nucleation and the nucleation rate drastically rises for at least 4 orders of magnitude when the width of nanowires decrease from 190 nm to 20 nm. Therefore, instead of constant density of nuclei used in the previous paper, here a time- and size-dependent nucleation rate is used:[5]

$$I = I^* \frac{d^*}{d} \exp(\frac{E_a^* - E_a^r}{kT}) \tag{S1}$$

with d the diameter of the NPs, E_a^r the activation energy for the NPs with a diameter of r, k the Boltzmann constant and T the temperature. The symbol with a star represent the values for the bulk materials (here the nanowires with 190 nm in width).[5] The values for activation energies at different sizes are taken from reference.[5]

Because of the relatively low T_c observed here, an Arrhenius relation was used for crystal growth rate:

$$u = u_0 \exp(-\frac{E_g}{kT}) \tag{S2}$$

with E_g the activation energy (2.4 eV).[6] Using JMAK theory,[7–11] we can derived the peak temperatures of phase transformation (identical as T_c defined in the present work). Adjusting the value of u_0 in Equation S2, the T_c of bulk GST is set to 150 °C, close to the value for bulk GST PCMs from literature. The modeled T_c as a function of diameter is displayed as the purple data points, in Figure 4 in main text.

References

1. Gammer, C., Mangler, C., Rentenberger, C. & Karnthaler, H. P. Quantitative local profile analysis of nanomaterials by electron diffraction. *Scr. Mater.* **63,** 312–315 (2010).
2. Tomforde, J. *et al.* Thin Films of Ge–Sb–Te-Based Phase Change Materials: Microstructure and in Situ Transformation. *Chem. Mater.* **23,** 3871–3878 (2011).
3. Ghezzi, G. E. *et al.* Crystallization of Ge2Sb2Te5 nanometric phase change material clusters made by gas-phase condensation. *Appl. Phys. Lett.* **101,** 233113 (2012).
4. Chen, B., Momand, J., Vermeulen, P. A. & Kooi, B. J. Crystallization Kinetics of Supercooled Liquid Ge–Sb Based on Ultrafast Calorimetry. *Cryst. Growth Des.* **16,** 242–248 (2016).
5. Lee, S.-H., Jung, Y. & Agarwal, R. Size-Dependent Surface-Induced Heterogeneous Nucleation Driven Phase-Change in Ge2Sb2Te5 Nanowires. *Nano Lett.* **8,** 3303–3309 (2008).
6. Kalb, J., Spaepen, F. & Wuttig, M. Atomic force microscopy measurements of crystal nucleation and growth rates in thin films of amorphous Te alloys. *Appl. Phys. Lett.* **84,** 5240–5242 (2004).
7. Avrami, M. Kinetics of Phase Change. I General Theory. *J. Chem. Phys.* **7,** 1103–1112 (1939).
8. Avrami, M. Kinetics of Phase Change. II Transformation-Time Relations for Random Distribution of Nuclei. *J. Chem. Phys.* **8,** 212–224 (1940).
9. Avrami, M. Granulation, Phase Change, and Microstructure Kinetics of Phase Change. III. *J. Chem. Phys.* **9,** 177–184 (1941).
10. Kooi, B. J. Monte Carlo simulations of phase transformations caused by nucleation and subsequent anisotropic growth: Extension of the Johnson-Mehl-Avrami-Kolmogorov theory. *Phys. Rev. B* **70,** 224108 (2004).

11. Kooi, B. J. Extension of the Johnson-Mehl-Avrami-Kolmogorov theory incorporating anisotropic growth studied by Monte Carlo simulations. *Phys. Rev. B* **73,** 54103 (2006).
